# Development of Healthy Vegan Bonbons Enriched with Lyophilized Peach Powder

**DOI:** 10.3390/foods11111580

**Published:** 2022-05-27

**Authors:** Dasha Mihaylova, Aneta Popova, Zhivka Goranova, Pavlina Doykina

**Affiliations:** 1 Department of Biotechnology, Technological Faculty, University of Food Technologies, 4002 Plovdiv, Bulgaria; dashamihaylova@yahoo.com; 2 Department of Catering and Nutrition, Economics Faculty, University of Food Technologies, 4002 Plovdiv, Bulgaria; pavlina_doikina@abv.bg; 3 Department of Food Technologies, Institute of Food Preservation and Quality-Plovdiv, Agricultural Academy, 4002 Plovdiv, Bulgaria; jivka_goranova@abv.bg

**Keywords:** health enhancing, raw snack, health claims, healthy ingredients

## Abstract

Changing nutritional demands, in combination with the global trend for snacking, sets a goal for preparing food products for direct consumption with certain beneficial properties. This study was designed to investigate the quality characteristics of raw vegan bonbons enriched with lyophilized peach powder. Three types of formulations were prepared in which 10%, 20%, and 30% of lyophilized peach powder were, respectively, added. The newly developed vegan products were characterized in terms of their physical (moisture, ash, color, water activity), microbiological, and nutritional characteristics. Their antioxidant activity, flavonoid, and phenolic content were also evaluated. Considering the content of the bonbons, the reported health claims indicate that they are sources of fiber, with no added sugar, and contain naturally occurring sugars. The color measurements demonstrated similarity in the values. This study showed that there is significant potential in the production of healthy snacks for direct consumption, with beneficial properties.

## 1. Introduction

Food choice may be influenced by many factors such as calories in meals, personal preferences, flavor, diet practice, etc. [1]. The eating behavior is directly linked to the food choices a person makes throughout his life cycle. The COVID-19 outbreak has reportedly led to unhealthy changes in eating patterns [2], which emphasizes the need to promote healthy habits and foods.

The effect of plant-based diets, with their diverse range of dietary patterns, has been extensively studied throughout the years [3]. They are widely associated with a reduced risk of noncommunicable diseases and early mortality [4]. Some attribute their rapid spread to the sustainability of the food production system, and the welfare of animals [5]. Whatever the primary reason, plant-based diets continue to grow in popularity.

Fruit and nuts are undoubtedly beneficial to one’s dietary intake. Fruits contain a palette of vitamins, minerals, and antioxidants [6], which provide obtainable advantages from their consumption, i.e., better cardiovascular health, improved response to some diseases, weight control, protective properties, etc. [7]. Nuts are usually integrated into daily diets because of their nutritional composition and health-promoting properties [8,9]. Their increased consumption, however, may lead to weight gain because of their fat content [10].

The “Evmolpiya” peach is a native Bulgarian late-season variety with health-promoting properties. It has a rich total phenolic content and total flavonoid content [11]. Moreover, its extracts showed potency to inhibit certain enzymes (α-glucosidase, lipase, α-amylase, and acetylcholinesterase) [12]. To date, it has not been extensively studied or used as an ingredient in culinary products, which makes the “Evmolpiya” peach an interesting object of research in the field.

All population groups eat between meals, which makes healthy snacking extremely important to maintaining good health [13]. Healthy snacks comprise the recommended nutrients and are associated with positive effects on the human body [14]. Scientists have presented and characterized several varieties of raw bars—with ingredients from the Amazon [15], papaya and tomato [16], sunroot, potato, and oat [17], or with added protein [18], which proves that this area of research is trending and presents endless opportunities for healthy nutrition [19].

Thus, the aim of the current research was to characterize and present raw vegan bonbons as healthy snack alternatives in the human diet. Three types of formulations were prepared in which 10%, 20%, and 30% of lyophilized peach powder were, respectively, added. The newly developed vegan products were characterized in terms of their physical (moisture, ash, color, water activity), microbiological, and nutritional characteristics. Their antioxidant activity, flavonoid, and phenolic content were also evaluated.

## 2. Materials and Methods

### 2.1. Materials

Fresh peach samples of the “Evmolpiya” variety were provided from the Fruit Growing Institute, Plovdiv, Bulgaria. The samples were lyophilized and powdered with a Tefal GT110838 grinder. Raw nuts and dried fruit were purchased from a local “Lidl” store (Plovdiv, Bulgaria). Both nuts and fruit are produced and packaged (200 g) by Lidl Stiftung & Co. KG., Neckarsulm, Germany. The raw cocoa butter was produced and packaged by “Dragon superfoods” and purchased at a local “dm drogerie” store in Plovdiv, Bulgaria.

### 2.2. Preparation of Bonbons

The bonbons were prepared in laboratory conditions at the University of Food Technologies. Table 1 provides information about the percentage distribution of the ingredients used to prepare the formulations.

**Figure 1:**
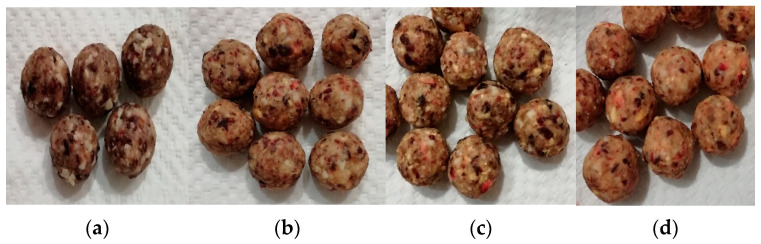
Bonbon formulations: (**a**) control sample; (**b**) LPP10; (**c**) LPP20; (**d**) LPP30.

The nuts and fruit were finely chopped with the use of Silver Crest chopper SMZ 260 J4 (260 W) at the turbo boost button speed for approximately 30 s. The cocoa butter was heated in a water bath in order to be poured over at the quantity needed. The ingredients were then hand-mixed until resulting in a soft plastic mass. In order to produce bonbons of similar size and weight, the soft mass was placed in a mold and after that was hand-rounded. The bonbons (Figure 1) were stored in a refrigerator upon their further usage.

### 2.3. Size and Weight Measurements

Weight was measured on a digital scale (KERN, EMB 1000-2). The diameter was measured with the use of a digital caliper SD-150.

### 2.4. Ash Content

Ash content was determined by burning in a muffle furnace according to AOAC 945.46 [20].

### 2.5. Moisture Content

The total moisture content of the samples was determined according to the procedure described in AACC method 44-15A [21].

### 2.6. Nutritional Data

The calculation method was used to determine the nutritional data. Supplier specifications for each of the ingredients (nuts, fruit, oil) were used to calculate the nutritional value of the finished products per 100 g. Information about the “Evmolpiya” peach was retrieved from previous research [11].

### 2.7. Color

A PCE-CSM 2 (PCE-CSM instruments, Deutschland) with a measuring aperture of 8 mm was used to analyze the color parameters. The L* (lightness; ranging from 0 to 100), a (representing red-green opponent colors), b (representing blue-yellow opponent colors), chroma (color saturation), and hue angle (color tone) were estimated.

### 2.8. Texture Profile Analysis (TPA)

TPA was performed by Texture Analyzer (Stable Microsystems, TAXT-2i Texture Analyzer, Godalming, UK), as described by Mazumder et al. [22]. The texture parameters (hardness, fracturability, maximum compressive force, and adhesiveness) were determined in a texture profile analysis mode (TPA) with speed before test 1.0 mm/s, trigger 5 g, speed after test 10 mm/s, voltage 60%, probe with a diameter 5 mm, distance 5 mm and specialized software “Texture Exponent“.

### 2.9. Determination of Total Polyphenolic Content (TPC)

An extraction procedure was performed to evaluate the total polyphenolic content, total flavonoid content, and antioxidant activity of the bonbon formulations. Specifically, 5 g of each formulation was subjected to extraction with 25 mL 96% ethanol at 25 °C and 200 rpm for 2 h. The mixtures were then centrifuged at 4000× *g* for 10 min, and the supernatant from each extraction was collected and used for analyses. The TPC was analyzed following a modified method of Kujala et al. [23], with some modifications [11]. The absorbance was measured at 765 nm and the TPC was expressed as mg gallic acid equivalents (GAE) per g dw.

### 2.10. Determination of Total Flavonoid Content (TFC)

The total flavonoid content was evaluated according to the method described by Kivrak et al. [24]. Results are expressed as μg QE/g dw, and quercetin (QE) was used as a standard.

### 2.11. Determination of Antioxidant Activity (AOA)

#### 2.11.1. DPPH^•^ Radical Scavenging Assay

The ability of the extracts to donate an electron and scavenge 2,2-diphenyl-1-picrylhydrazyl (DPPH) radical was determined by the slightly modified method of Brand-Williams et al. [25] as described by Mihaylova et al. [26]. The DPPH radical scavenging activity was presented as a function of the concentration of Trolox—Trolox equivalent antioxidant capacity (TEAC)—and was defined as the concentration of Trolox having equivalent antioxidant activity expressed as μM TE/g dw.

#### 2.11.2. ABTS^•+^ Radical Scavenging Assay

The radical scavenging activity of the extracts against 2,2′-azino-bis(3-ethylbenzothiazoline-6-sulfonic acid) (ABTS^•+^) was estimated according to Re et al. [27]. The results are expressed as TEAC values (μM TE/g dw).

#### 2.11.3. Ferric-Reducing Antioxidant Power (FRAP) Assay

The FRAP assay was carried out according to the procedure of Benzie and Strain [28]. The results are expressed as TEAC values (μM TE/g dw).

#### 2.11.4. Cupric-Ion-Reducing Antioxidant Capacity (CUPRAC) Assay

The CUPRAC assay was carried out according to the procedure of Apak et al. [29]. Trolox was used as a standard, and the results are expressed as TEAC values (μM TE/g dw).

### 2.12. Water Activity

The water activity (a_w_) was assessed using a Rotronic HP23-AW-A Lachen, Bassersdorf, Switzerland.

### 2.13. Microbial Count—Product Shelf Life

Bonbons were tested using the spread-plate method on days 1, 3, and 5 of storage to determine yeasts and molds (YM) using potato dextrose agar. Potato dextrose agar plates were incubated at 30 °C and counted after 72 h. The aerobic mesophilic microorganisms (AMM) count was evaluated according to ISO 4833-1:2013 [30] using plate count agar as a culture medium. The results are expressed as colony-forming units (CFUs)/mL.

### 2.14. Microscopic Imaging

The photographs of the surface of the bonbon were produced via a USB digital pocket microscope MX200-B, with a 1000× LED magnification endoscope camera and a focus range of 1–9 mm.

### 2.15. Statistical Analysis

MS Excel software was used for data analysis. All assays were performed in at least triplicates. Results are presented as mean ± SD (standard deviation). Relevant statistical analyses of the data were presented using one-way ANOVA and a Tukey–Kramer post hoc test (α = 0.05), as described by Assaad et al. [31].

## 3. Results and Discussion

In order to characterize the bonbons, they were first evaluated in terms of their moisture and ash content, as well as their size (diameter, mm) and weight (Table 2). After that, the proximate nutritional data were gathered, which are presented in Table 3.

The different formulations had relatively the same moisture content, but LPP20 was the most similar to the control sample. When the ash content is considered, again no significant differences were observed, and all three enriched formulations had relatively analogous values. These values correspond well to the ones reported in the literature concerning the ash and moisture content of nuts [32], a major component in the formulations.

**Figure 2:**
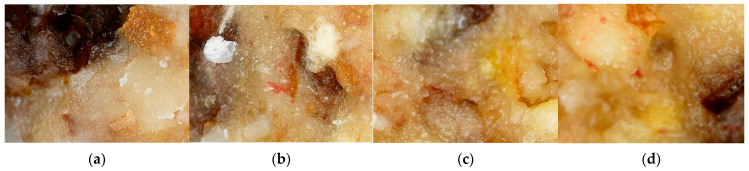
Electronic microscopic photographs of bonbon formulations’ surface (160×): (**a**) control sample; (**b**) LPP10; (**c**) LPP20; (**d**) LPP30.

In terms of weight and size, no notable differences exist to the naked eye. Hand-made products are usually very difficult to prepare in order to make them visibly the same. Even though the products were thoroughly mixed prior to shaping, it has to be noted that different ingredients can exist in different quantities. A 160× magnification of the bonbon’s surface proves this point (Figure 2). The micrographs very clearly show parts of the dried fruit, cocoa butter, and microscopic peach particles. Parts of the nuts can also be recognized.

Raw bars are versatile, ready-to-eat products that are highly appreciated for their convenience and healthy nutrients [33]. The currently prepared formulations can be related to raw bars, with the exception of their shape.

Considering the content of the bonbons, health claims reported in regulation (EC)1924/2006 [34] of the European Parliament and the Council of 20 December 2006 state that they are sources of fiber (at least 3 g fiber per 100 g), with no added sugar, and contain naturally occurring sugars.

Concerning the protein content, it ranged from 3.89 g/100 g (LPP30) to 7.77 g/100 g (control sample). The main contributors to the protein content are the nuts present in the formulations. The products cannot be considered sources of protein, as they do not provide enough to cover the daily needs of healthy individuals. For example, in order to provide enough protein in snack bars, other authors have used whey protein isolate or whey protein concentrate as an ingredient [18,35].

Regarding carbohydrates, the inclusion of more lyophilized peach powder (such as in LLP30) led to the lowering of the sugar content by 1.8 times. Carbohydrates in the formulations are mostly due to the fruit content of the formulations—dried cranberries and raisins. Compared to some of the commercially available raw bars in which the average content is 40 g/100 g, the currently presented formulations are more favorable in terms of sugar intake and energy provided from carbohydrates. 

Regarding the lipid content, it varied from 45.21 to 50.54 g/100 g. This content is approximately 30% higher than the commercially available bars. The current lipid content is also higher than the one reported for high-protein bars [18]. However, compared to the control sample, it can be seen that the incorporation of lyophilized peach powder contributed to the lowering of the lipid content by 5%. It has to be noted, however, that there was a 13% increase in the monosaturated fat content in the LPP30 formulation, compared to the control. 

The amount of energy obtained by consuming 100 g of the bonbon formulations will contribute significantly to the daily energy intake of healthy individuals. The energy value of formulation LPP30 was the most similar to commercially available raw bars. All of the formulations have a higher energy count than raw bars presented by other researchers [18]. It has to be mentioned that the lipid content is often excluded from the data presented in some papers, so a comparison is not always possible. This can be seen as a limitation for providing a broader discussion.

Color is an important determinant when it comes to deciding if a food is appealing and desirable to eat. Thus, the CIELAB color spectra of the studied formulations were determined, which are presented in Table 4.

The color of the formulations was formed by the ingredients present in them, and no artificial colorants were used. The highest values for brightness belonged to the LPP20 formulation. The “h” value ranging from 62.44 ± 1.89 (LPP10) to 64.98 ± 1.55 (LPP20) suggested the presence of an orangey shade. This is seen very well in the micrographs in Figure 2. Natural colorants are commonly observed by lower “c” values and higher L values [36], which is supported by the current results, suggesting that the formulations are interpreted as natural in color. Some authors propose that the lightness can increase with the addition of more fruits and dry ingredients in general [37]. This proposition is supported in the current study, as the L value of the control was lower than the L value of the formulations with added peach powder. 

All formulations, including the control sample, did not have significant differences in the measured parameters, which can indicate that certain consumers will perceive the formulations as similar or the same in color. However, the calculated ∆E value for formulations LPP10 (8.49), LPP20 (14.89), and LPP30 (14.99) suggests that the human eye should perceive a difference between the control sample and the newly developed ones, although LPP20 and LPP30 were indeed very similar. Limited or no data about the color spectra of other raw bars exist; thus, a comparison was not applicable. Moreover, in order to make a parallel comparison, similar ingredients should be used.

The TPC content of the formulations is presented in Table 5. The TPC showed the highest value in the control sample and the lowest value in LPP30 in which 30% of lyophilized peach powder was present. This hints at the fact that nuts and other fruit (raisins and cranberries) might contribute more to the TPC content. All of the ingredients used were proven to contain health-promoting phenolic compounds [38,39]. 

When the TFC is considered (Table 5), LPP20 was the formulation with the highest values. Here, again, the heterogenic distribution of the ingredients found in the bonbon might lead to the established results, since all nuts and fruit parts of the formulation were found to contain certain flavonoids. Dried fruits, in general, are valuable sources of bioactive compounds, i.e., flavonoids [40]. 

**Figure 3:**
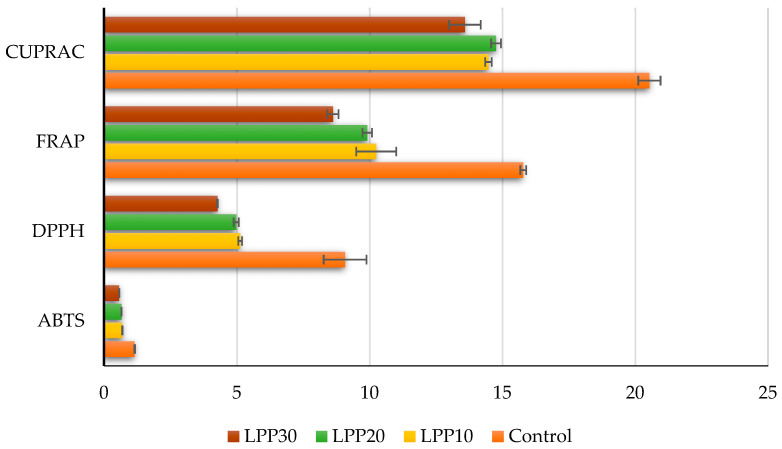
Antioxidant properties of bonbon formulations: ABTS—mMTE/g dw; DPPH, FRAP, CUPRAC—µMTE/g dw.

A strong association between polyphenols and antioxidant properties exists [41,42,43,44]. The formulations were subjected to antioxidant analysis using ABTS, DPPH, FRAP, and CUPRAC methods in order to gain a better understanding of their antioxidant activity (Figure 3).

As seen in Figure 3, the control sample showed the most promising results in all assays. The highest values were reported in the ABTS assay (0.57–1.15 mMTE/g dw). All formulations containing lyophilized peach powder showed similar results, which may lead to the conclusion that the percentage incorporated does not influence the overall antioxidant activity. 

Texture is an important multi-parameter property that is commonly used for food quality control [45]. Table 6 provides a visual presentation of the formulations’ texture profile analysis on days 1 and 5 of their production, in terms of their hardness, fracturability, maximum compressive force (MCF), and adhesiveness. Variations in the shape and size of the ingredients used for the formulations, as well as the interaction they initiate in their preparation, can justify the relatively high observed standard deviations for some of the parameters assessed by the texture analyzer [46].

The hardness of the bonbon formulations increased progressively for 5 days of storage. The presence of more sugars usually causes increased hardness [47]. This is not supported by the current results according to which LPP30 had the least amount of sugar, and the control sample had the most. The highest value of hardness was recorded for LPP30, which contained higher amounts of lyophilized peach powder and cocoa butter. The lowest level of hardness was recorded in the control sample, in which no peach powder was present. Moisture migration may be responsible for the increased hardness, because it is induced by the formation of bonds between sugars and proteins, and moisture acts as a plasticizer and reduces the formation of this bond [48]. Thus the less the moisture present in the sample the more the hardness values. This is seen very well in formulation LPP10 in which there is the least amount of moisture (%), compared to the other formulations, and the greatest change in the hardness for 5 days of storage—1.3 times.

Hardness, fracturability, and MCF were reported separately, as each sample behaved differently during compression. On day 1, all samples disintegrated prior to 60% stress but retained MCF. On day 5, all samples required more force to initiate fracture than compression at 60% stress. The force required to break was significantly affected by the ingredients used. 

LPP30 remained more adhesive than the control on day 1. An increase in the adhesion of all samples during storage was observed. The greatest change was recorded for LPP20. The smallest change in adhesion was noted in the control sample—50%. 

Water activity is an important factor that is directly linked to the shelf life of food products. The water activity of the bonbons was evaluated on days 1 and 5 of their production, and the established results are presented in Table 7.

A tendency toward reduced water activity during bonbon storage was observed. A higher value for water activity in the control sample may be due to its higher moisture content. Neves [49] reports that fruit bars with added protein, high in carbohydrates, have higher water activity due to the higher moisture content, which decreases during storage. Additionally, the a_w_ of the bonbons varied depending on the components and the storage period. According to Silva et al. [50], foods with intermediate humidity usually have an a_w_ from 0.9 to 0.6, which is low enough to keep the product from microbial spoilage and ensure its stability. Water activity in the range of 0.65 to 0.75 contributes to shorter food life due to intermediate humidity levels. Outside this range, products can be stored for a longer period of time [51]. Similarly, the control sample and formulations LPP20 and LPP30 showed higher reductions in water activity during 5 days of storage.

The currently stated values correspond well to the ones reported for cereal bars, ranging from 0.557 to 0.597 [52]. Other authors [53] also document similar a_w_ values for sweet-cherry, almond, and honey snack bars (0.467–0.508). This suggests that the developed formulations are favorable in terms of microbial growth inhibition. 

**Figure 4:**
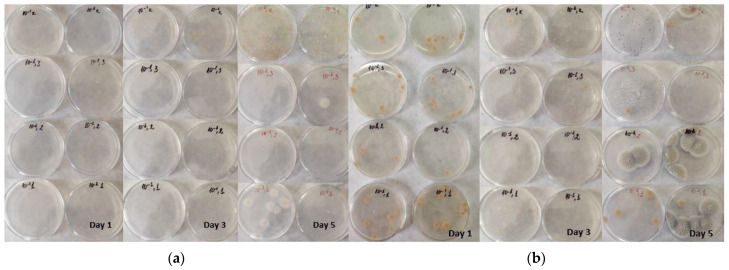
View of microbial growth in bonbon formulations: (**a**) AMM; (**b**) YM.

The microbial load of the studied formulations is presented in Table 8 and Figure 4. All formulations can be considered safe for consumption, although the control sample is the most contaminated one during storage. This indefinitely hints that the introduced lyophilized peach powder contributes well to several quality parameters including microbial load.

On day one, the highest plate count was noted in the control sample, and the lowest value in LPP20, while the mold count of the formulations varied from 500 to 1500 CFU/mL. The observed differences are most probably due to the difference in major ingredients part of the recipe.

The inclusion of a higher amount of lyophilized peach powder (LPP20 and LPP30) led to a decreased plate count on day 3; in addition, on day 5, a plate count similar to the control on day 1 was observed. This could be due to the small particles of peach powder drawing moisture from the product and leaving less water available for microbial activity [54] and to the antimicrobial properties of the “Evmolpiya” peach itself. Microorganisms are mostly neutrophilic and cannot grow at less than 4.5 pH and 0.8 water activity [35]. Lower water activity, in general, helps to prevent the proliferation of microorganisms [55]. Fruits usually have acidic pH and contribute well in this regard; the pH registered for the “Evmolpiya” variety is 3.65 [56]. Furthermore, the reported water activity for all formulations was below 0.8. 

There are various factors that influence mold growth, including water activity, relative humidity, temperature, pH, and storage time. Mold count decreased for all formulations with lyophilized peach powder on day 5. This may be induced by the low a_w_, bearing in mind that the survival of mold at lower a_w_ depends on various factors, i.e., nutrient availability, temperature, and pH [55]. Sugar concentrations also aid in the inhibition of mold growth in the formulations [57].

## 4. Conclusions

The results of this research revealed the possibility of developing raw vegan bonbons with the addition of lyophilized peach powder. 

Considering the content of the bonbons, the reported health claims indicate that they are sources of fiber, with no added sugar, and contain naturally occurring sugars. Color measurements demonstrated similarity in the values. When it comes to texture, the hardness of the bonbon formulations increased progressively for 5 days of storage. A_w_ decreased, and the microbial load showed that the lyophilized peach powder as an ingredient has a favorable influence on the plate count and mold growth.

The TPC results showed the highest value in the control sample and the lowest value in LPP30. When the TFC is considered, LPP20 was the formulation with the highest values. All formulations containing lyophilized peach powder showed similar AOA results, which may lead to the conclusion that the percentage incorporated does not influence the overall antioxidant activity. 

The newly developed bonbon formulations can be used as a quick snack throughout the day or as enrichment to one’s daily healthy meal plan. Further research can pave a way for the incorporation of protein-added ingredients in these formulations.

## Figures and Tables

**Table 1:** Bonbon formulations: LPP—lyophilized peach powder.

Type of Bonbon	Walnut, %	Almond, %	Raisin, %	Cranberry, %	Cocoa Butter, %	LPP, %
Control	18	18	18	18	28	-
LPP10	15	15	15	15	30	10
LPP20	12	12	12	12	32	20
LPP30	9	9	9	9	34	30

**Table 2:** Weight (g), size (mm), ash (%), and moisture (%) content of bonbons.

Bonbon Formulations	Weight, g	Diameter, mm	Ash Content, %	Moisture Content, %
Control	8.87 ± 0.60 ^a^	25.15 ± 0.39 ^a^	1.20 ± 0.31 ^a^	7.51 ± 0.03 ^a^
LPP10	8.31 ± 0.54 ^a^	25.12 ± 0.67 ^a^	1.44 ± 0.34 ^a^	5.05 ± 0.05 ^d^
LPP20	7.92 ± 0.69 ^a^	24.61 ± 0.88 ^a^	1.41 ± 0.08 ^a^	7.07 ± 0.09 ^b^
LPP30	7.86 ± 0.79 ^a^	24.70 ± 0.80 ^a^	1.47 ± 0.00 ^a^	6.45 ± 0.06 ^c^

Different letters in the same column indicate statistically significant differences (*p* < 0.05), according to ANOVA (one-way) and the Tukey test.

**Table 3:** Nutritional data of bonbon formulations.

Bonbon Formulations, 100 g	Proteins, g	Carbohydrates, g	Sugars, g	Fiber, g	Fat, g	Monosaturated Fats, g	ώ 3, g	Energy, kcal
Control sample	7.77	29.16	25.70	4.96	50.54	18.96	1.73	612.36
LPP10	6.48	25.50	21.89	4.38	48.76	19.80	1.44	574.99
LPP20	5.18	21.87	18.09	3.80	46.98	20.64	1.15	537.63
LPP30	3.89	18.22	14.27	3.22	45.21	21.48	0.86	500.27

**Table 4:** CIELAB color spectra of bonbon formulations.

Bonbon Formulations	L	a	b	c	h
Control sample	47.74 ± 4.26 ^a^	9.46 ± 1.97 ^a^	15.66 ± 2.33 ^b^	18.44 ± 1.63 ^b^	58.63 ± 8.13 ^a^
LPP10	50.88 ± 2.13 ^a^	11.38 ± 1.31 ^a^	21.75 ± 1.19 ^ab^	24.56 ± 1.57 ^ab^	62.44 ± 1.89 ^a^
LPP20	54.96 ± 2.65 ^a^	13.12 ± 1.62 ^a^	28.04 ± 2.01 ^a^	30.96 ± 2.44 ^a^	64.98 ± 1.55 ^a^
LPP30	53.85 ± 2.50 ^a^	12.42 ± 1.67 ^a^	26.57 ± 1.83 ^a^	29.39 ± 1.21 ^a^	64.86 ± 4.22 ^a^

Different letters in the same column indicate statistically significant differences (*p* < 0.05), according to ANOVA (one-way) and the Tukey test.

**Table 5:** Total flavonoid content (TFC) and total phenolic content (TPC) of bonbon formulations.

Bonbon Formulations	Total Flavonoid Content, μgQE/g fw	Total Phenolic Content, mgGAE/g dw
Control sample	84.64 ± 1.69 ^c^	1.89 ± 0.03 ^a^
LPP10	78.13 ± 1.36 ^d^	1.33 ± 0.00 ^c^
LPP20	117.63 ± 1.37 ^a^	1.40 ± 0.04 ^b^
LPP30	100.29 ± 2.55 ^b^	1.21 ± 0.01 ^d^

Different letters in the same column indicate statistically significant differences (*p* < 0.05), according to ANOVA (one-way) and the Tukey test.

**Table 6:** Texture profile analysis of bonbon formulations.

Bonbon Formulations	Hardness/MCF, N		Fracturability, N		Adhesiveness, J	
	Day 1	Day 5	Day 1	Day 5	Day 1	Day 5
Control	31.14 ± 1.96 ^a^	38.03 ± 3.11 ^a^	16.10 ± 2.26 ^a^	18.02 ± 0.51 ^a^	0.18 ± 0.05 ^a^	0.36 ± 0.09 ^a^
LPP10	33.89 ± 4.79 ^b^	42.80 ± 2.80 ^b^	19.88 ± 1.92 ^a^	19.46 ± 1.28 ^a^	0.32 ± 0.03 ^b^	0.73 ± 0.07 ^b^
LPP20	41.90 ± 2.93 ^c^	46.81 ± 0.62 ^c^	22.25 ± 1.22 ^ab^	28.42 ± 6.47 ^b^	0.35 ± 0.04 ^b^	1.14 ± 0.63 ^c^
LPP30	48.94 ± 1.79 ^d^	54.02 ± 2.94 ^d^	24.91 ± 2.27 ^c^	30.50 ± 1.39 ^b^	0.38 ± 0.03 ^c^	0.98 ± 0.15 ^d^

Different letters in the same column indicate statistically significant differences (*p* < 0.05), according to ANOVA (one-way) and the Tukey test.

**Table 7:** Water activity (a_w_) of bonbon formulations.

Bonbon Formulations	Water Activity, a_w_	
	Day 1	Day 5
Control sample	0.559 ± 0.007 ^c^	0.546 ± 0.06 ^c^
LPP10	0.503 ± 0.009 ^b^	0.496 ± 0.06 ^b^
LPP20	0.492 ± 0.003 ^a^	0.482 ± 0.003 ^b^
LPP30	0.468 ± 0.013 ^a^	0.458 ± 0.013 ^a^

Different letters in the same column indicate statistically significant differences (*p* < 0.05), according to ANOVA (one-way) and the Tukey test.

**Table 8:** Microbial count of bonbon formulations: YM—yeasts and molds; AMM—aerobic mesophilic microorganisms.

Bonbon Formulations	YM, CFU/mL			AMM, CFU/mL		
	Day 1	Day 3	Day 5	Day 1	Day 3	Day 5
Control sample	1500	2100	3600	1050	3400	25,000
LPP10	1150	2200	1450	400	1000	2250
LPP20	500	700	600	100	950	1000
LPP30	1300	1000	500	600	200	1100

## Data Availability

The data presented in this study are available on request from the corresponding author.
